# Differences in primary health care delivery to Australia’s Indigenous population: a template for use in economic evaluations

**DOI:** 10.1186/1472-6963-12-307

**Published:** 2012-09-07

**Authors:** Katherine S Ong, Rob Carter, Margaret Kelaher, Ian Anderson

**Affiliations:** 1 Centre for Health Policy, Programs and Economics, School of Population Health, The University of Melbourne, Carlton, Victoria 3010, Australia; 2 Deakin Health Economics, Deakin Strategic Research Centre – Population Health, Deakin University Public Health Research Evaluation and Policy Cluster, Deakin University, Burwood Highway, Burwood, Victoria 3125, Australia; 3 Murrup Barak, Melbourne Institute for Indigenous Development, The University of Melbourne, Carlton, Victoria 3010, Australia

**Keywords:** Health economics, Resource allocation, Indigenous Australians, Primary health care services, Health service delivery, Health equity

## Abstract

**Background:**

Health economics is increasingly used to inform resource allocation decision-making, however, there is comparatively little evidence relevant to minority groups. In part, this is due to lack of cost and effectiveness data specific to these groups upon which economic evaluations can be based. Consequently, resource allocation decisions often rely on mainstream evidence which may not be representative, resulting in inequitable funding decisions. This paper describes a method to overcome this deficiency for Australia’s Indigenous population. A template has been developed which can adapt mainstream health intervention data to the Indigenous setting.

**Methods:**

The ‘Indigenous Health Service Delivery Template’ has been constructed using mixed methods, which include literature review, stakeholder discussions and key informant interviews. The template quantifies the differences in intervention delivery between best practice primary health care for the Indigenous population via Aboriginal Community Controlled Health Services (ACCHSs), and mainstream general practitioner (GP) practices. Differences in costs and outcomes have been identified, measured and valued. This template can then be used to adapt mainstream health intervention data to allow its economic evaluation *as if* delivered from an ACCHS.

**Results:**

The template indicates that more resources are required in the delivery of health interventions via ACCHSs, due to their comprehensive nature. As a result, the costs of such interventions are greater, however this is accompanied by greater benefits due to improved health service access. In the example case of the polypill intervention, 58% more costs were involved in delivery via ACCHSs, with 50% more benefits. Cost-effectiveness ratios were also altered accordingly.

**Conclusions:**

The Indigenous Health Service Delivery Template reveals significant differences in the way health interventions are delivered from ACCHSs compared to mainstream GP practices. It is important that these differences are included in the conduct of economic evaluations to ensure results are relevant to Indigenous Australians. Similar techniques would be generalisable to other disadvantaged minority populations. This will allow resource allocation decision-makers access to economic evidence that more accurately represents the needs and context of disadvantaged groups, which is particularly important if addressing health inequities is a stated goal.

## Background

With growing health expenditures and greater scrutiny of health care spending, the results of economic evaluations are increasingly used to assist decisions about health care resource allocation [1]. Economic evaluations assist decision-makers to determine which health interventions represent the best ‘value for money’ in terms of maximizing the health of the population with the available funds.

Such economic evidence is valuable, particularly for groups who experience health disadvantage such as Australia’s Aboriginal and Torres Strait Islander (or Indigenous) population, to indicate where resources are best placed to help bridge the health gap. Australia’s Indigenous population has a health status much worse than that of the general Australian population, with life expectancy 10 years below that of non-Indigenous Australians [2], and standardized morality and infant mortality rates more than twice as high [3]. This poorer health is grounded in complex historical, geographic, economic and socio-cultural factors, in many ways similar to those faced by colonized indigenous peoples worldwide. The available evidence suggests that mainstream primary health care services have struggled to deal with these issues and improve the Indigenous health discrepancy, and that population specific services are warranted [4-7].

It is generally accepted within Australian policy discourse that greater resources are necessary to improve Indigenous health [4,8,9]. Qualitative evidence suggests that ‘best practice’ primary health care for the Indigenous population is based on self-determination and community control, epitomized by the Aboriginal Community Controlled Health Service (ACCHS) model of comprehensive primary health care [10-12]. An ACCHS is defined as being an incorporated Aboriginal organization, initiated and based in a local Aboriginal community, governed by a locally elected Aboriginal body, and delivering a holistic and culturally appropriate health service to the community that controls it [13]. Community control is a central component of this model; in other words, the health service is run by Aboriginal people, for Aboriginal people. Based on the premise of providing ‘equity of access’, ACCHSs have been found to provide equitable and more effective primary health care for the Indigenous population [11]. However, the funding of ACCHSs remains fragmented and is generally considered insufficient to meet the greater health need [14].

The results of economic evaluations could help determine the best use of resources to improve Indigenous health. However, such evidence specific to the Indigenous context remains deficient. In part, this can be explained because economic evaluation techniques depend on modeling from existing quantitative data, to determine both the total costs of a health intervention, and to extrapolate to improvements in health effect or benefit [15]. Due to the relatively small size of Australia’s Indigenous population, there is a lack of cost and effectiveness data specifically pertaining to this group that takes into account their unique demographic features, socio-cultural context, and preferred health service models. Therefore, there is a corresponding lack of Indigenous specific health economics data. This means that resource allocation decisions for Indigenous health are often based on mainstream economic evidence which may not be representative, or not based on economic evidence at all. Under these circumstances cost-effectiveness results may be distorted, and health inequalities may in fact be perpetuated rather than improved.

This paper describes one method by which this deficiency in Indigenous health economics data could be overcome. The ‘Indigenous Health Service Delivery (IHSD) Template’ has been developed, which quantifies the differences in how health interventions are delivered to the Indigenous population via ACCHSs compared to mainstream general practitioner (GP) based services, the latter being the standard form of primary health care available in Australia. Differences in costs and benefits have been identified, measured and valued in the construction of the template. The IHSD Template can then be applied to adapt mainstream data, to allow its economic evaluation *as if* interventions were delivered to the Indigenous population via best practice methods of health service delivery. Therefore, economic evaluation results which are based on mainstream evidence can be made more relevant to the Indigenous context and facilitate more meaningful resource allocation recommendations.

An additional advantage of the IHSD Template is that it provides a measure of equitable health service provision, in terms of the additional costs incurred and the improved health benefits that result. Therefore, it is an important potential tool for decision-makers when achieving health equity is a pressing policy imperative, in an area where, to date, quantitative data has been limited.

The research outlined in this paper comprised part of the larger Assessing Cost Effectiveness in Prevention (ACE-Prevention) project, which evaluated the cost-effectiveness of interventions to prevent chronic disease in the Australian population [16]. As part of this study, separate economic evaluations were performed for the general (or total) Australian population and for the Indigenous Australian population. Separate evaluations were necessary to account for differences in demographics, the target disease burden, the prevalence and distribution of harmful exposures, the way health interventions are delivered, and their effectiveness. The content of this paper draws on the Indigenous component of the ACE-Prevention project, and expands on information contained within the ACE-Prevention final report and dissemination pamphlets [16,17].

## Methods

Mixed methods were used in the development of the IHSD Template. Data was sought to identify, measure and value the differences in how primary health care interventions are delivered between ACCHSs compared to mainstream GP services. Information was initially obtained from a general search of the publicly available literature. Searches were made of PubMed and Web of Science, along with the grey literature and discussion with experts in the field. This search only revealed a small number of studies, none of which provided sufficient quantitative information for use in economic evaluation [18,19]. Aggregated government data was available on levels of ACCHS service provision in the form of the Service Activity Reports [20], however no cost information was provided. Using the data gathered from the literature, a base IHSD Template was constructed.

Following the literature search, further information and validation of the data gathered was sought from the ACE-Prevention Indigenous Steering Committee (ISC), which consisted of academics and policy makers selected to represent a broad range of expertise in Indigenous health. Three key meetings were held between 2006 and 2009, during which the base IHSD Template was discussed, and feedback used to identify additional sources of information and improve the template structure.

In addition, key informant interviews were conducted with people working within the ACCHS sector. Sixteen interviews with staff of varying roles from a range of ACCHSs in different remoteness localities took place between July and November 2008 (Table 1). In total, staff from 5 different health services were represented. Informed consent was obtained in all cases and prior to the interview, a plain language summary of the IHSD Template and its structure was provided. This was followed by an interview using a standardized, semi-structured open-ended interview schedule that consisted of opinion and knowledge questions about how well the IHSD Template reflected real life work practices. In particular, the questionnaire was designed to validate the base IHSD Template that had been derived from the literature, and to obtain quantitative estimates of certain parameters that were not able to be found elsewhere. Ethics approval was obtained from the Human Research Ethics Committees of the University of Melbourne and Central Australia (via the Menzies School of Health Research). Subject recruitment was by snowball methods with initial identification made by research contacts within Indigenous health. The management of the ACCHSs were approached for initial permission to conduct the interviews, and feedback was in the form of a community report and dissemination of other project reports and publications [17].

**Table 1:** Summary of key informant characteristics

**Health service location**	**Total no. key informants**	**Position within health service**				
		**Manager**	**Medical**	**Nursing**	**AHW**	**Other**
Urban – Melbourne	**7**	3	2		2	
Rural - Victoria	**1**		1			
Remote – Central Australia and Northern Territory	**8**	1	4	2		1
**Total**	**16**	**4**	**7**	**2**	**2**	**1**

AHW = Aboriginal Health Worker.

Interviews were de-identified prior to analysis, and incorporated along with feedback from the ISC meetings and data from the literature to validate and further refine the template. Specifically, general comments regarding the IHSD Template were incorporated where possible. Responses to the quantitative questions were collated in a crude manner as estimates and used as inputs for the template where alternative data was not available. Ranges were used where relevant to reflect the diversity of key informant responses. Specific parameters for which information from the interviews was used are detailed in Additional File 1.

The IHSD Template describes differences in the conduct of standard patient consultations between ACCHSs and mainstream GP practices in terms of their costs and impact on treatment effects. In keeping with the terms of reference for the ACE-Prevention project, a health sector perspective has been taken, and all costs measured in Australian dollars and referenced to the year 2003 [21]. IHSD Template values have been calculated for remote area, non-remote area, and national average decision contexts, to account for regional differences in costs and service provision. The mainstream GP comparator is based on the provision in non-remote services, due to the relatively small proportion of non-Indigenous Australians who live in remote areas and the corresponding lack of remote mainstream GP practices.

Use of the IHSD Template has been trialled in several economic evaluations of interventions to prevent either cardiovascular disease, diabetes or chronic kidney disease in the Australian Indigenous population as part of the ACE-Prevention project [16]. This has involved the adaptation of disease-specific epidemiological models to ensure data is relevant to the Indigenous population. In this paper, the ‘Cardiovascular Disease Prevention Model’ has been used for the general Australian population, which was originally developed by Stephen Lim for the ACE-Heart disease project and subsequently used in the ACE-Prevention project [22-24]. This model is a Markov model developed in Microsoft Excel (Microsoft Corporation, Washington, USA) which uses the modified disability-adjusted life-year (DALY) as the measurement of health benefit [1]. The Cardiovascular Disease Prevention Model has been adapted to the Indigenous population as the ‘Indigenous Cardiovascular Disease Prevention Model’ by the author (KO, unpublished) to account for differences in the Indigenous burden of disease and demographic factors.

## Results

### Identification of IHSD Template components

Using the data collection methods described, a framework of ‘components’ have been identified which describe how health interventions delivered from ACCHSs differ from those delivered from mainstream GP services. Health service definitions were taken from the National Aboriginal Health Strategy [11] for ACCHSs and the Royal Australian College of General Practitioners [25] for GP services. The IHSD Template components are grouped into six broad categories as listed below (reproduced from Vos et al. [16] and Ong [17]):

***Basic health intervention delivery characteristics***:

• Role substitution – a patient may be seen by an Aboriginal Health Worker or a nurse in addition to, or instead of, a doctor

• Compliance management – e.g. medication dosing and appointment recalls

• Staff training activities – e.g. cultural in addition to professional training for non-Indigenous staff

• Emphasis on home visits

• Time spent on paperwork, case conferencing and the management of complex medical conditions

• Seeing of other family members as part of routine consultations

***Population health, social and community activities***:

• Provision of other services – e.g. social work and counseling

• Provision of services usually provided by outside agencies – e.g. pharmacy, financial and housing assistance

• Health promotion and community development activities

• Provision of a community space

***Management and governance structures***:

• Presence of a community management board and the associated need for community capacity building in management

• Additional management resources required for overseeing larger staff numbers and multiple projects

***Patient transport services*:**

• Provision of transport for patients to and from appointments

***Provision of services to a large remote population*:**

• Out of hours emergency care

• Outreach services

• Housing and relocation costs for staff

• Additional costs associated with pharmaceutical and pathology services

***Differences in rates of Indigenous utilization of services and adherence to treatments*:**

• Differences in rates when interventions are delivered from ACCHSs compared to mainstream GP clinics due to improved access

### Measurement and valuation of IHSD Template components

Differences in the quantity or magnitude of the IHSD Template components listed above (between ACCHSs and mainstream GP services) have been estimated based on the same sources of information outlined in the methods section. Where relevant, the components have also been valued in terms of cost. Total costs have then been calculated by multiplying the quantity of the component by its unit cost. Average additional costs are attributed to a single ‘encounter’ (standard consultation) with a health care practitioner at an ACCHS (Table 2). This then allows intervention costs to be adjusted according to the number of health practitioner visits that are involved in the ‘event pathway’ for an intervention. Therefore, additional costs of intervention delivery via ACCHSs are allocated as a form of joint cost (analogous to overhead costs) proportional to its health service requirements.

**Table 2:** **The additional cost of IHSD Template components (averaged across total Indigenous population) (Reproduced from Ong **[17]**) **

**IHSD Template component category**	**Additional cost per ACCHS patient encounter**
Basic health intervention delivery characteristics	$16.67-$31.57 (depending on consultation length)
Population health, social and community activities	$9.28
Management and governance	$3.87
Patient transport services	$47.01
Services to remote regions	$5.50

The component costs are totalled and presented in Table 3 as the ‘IHSD Template values’, along with the differences in rates of Indigenous utilization of health services, adherence to treatments, and the ratio of differences in future cost-offsets. Subgroup analysis has also been performed according to whether ACCHSs are located in remote or non-remote areas, and these results are also shown in Table 3. Further details of the data sources used in generating these values are available as Additional file 1.

**Table 3:** IHSD Template values for total Indigenous population (including 95% uncertainty ranges)

**IHSD Template value**	**Mainstream GP services (95% uncertainty)**	**ACCHS (95% uncertainty)**		
		**Total indigenous pop**	**Non-remote indigenous pop**	**Remote indigenous pop**
**Short consultation cost**	$ 30.85	$ 113.18 ($78.74–$149.43)	$ 91.21 ($59.93–$127.80)	$ 168.36 ($104.00–$235.42)
**Long consultation cost**	$ 58.55	$ 155.78 ($101.76–$220.13)	$ 129.34 ($76.99–$187.94)	$ 223.42 ($134.49–$319.30)
**Pathology test adjustment ratio**	1	1.16 (1.13–1.18)	1	1.60 (1.51–1.69)
**Indigenous utilization rate (cf. non-Indigenous)**	60.0% (50.8%–69.6%)	73.2% (63.0%–82.6%)	As for total Indigenous pop*	As for total Indigenous pop*
**Indigenous adherence rate (cf. non-Indigenous)**	77.8% (65.7%–89.7%)	95.7% (83.5%–108.3%)	As for total Indigenous pop*	As for total Indigenous pop*
**Cost-offsets ratio (Indigenous : non-Indigenous)**	1.19 (0.96–1.49)	1.19 (0.96–1.49)	As for total Indigenous pop*	As for total Indigenous pop*

* Indigenous utilization and adherence rates and cost-offsets were not assessed separately for the non-remote and remote populations, so values calculated are those for the total Indigenous population.

Uncertainty analysis has been included to account for uncertainty in the input parameters. A range of IHSD Template values have been generated by means of Monte Carlo simulation (2000 iterations) using the Excel add in software @Risk (Palisade Corporation, New York, USA), and this is displayed as uncertainty ranges in Table 3. These IHSD Template values can subsequently be used to adapt mainstream evidence for use in the economic evaluation of interventions from Indigenous settings.

### Use of the IHSD Template values in an example

Prior to discussing the IHSD Template, its use is best illustrated in an example economic evaluation. The intervention is the hypothetical application of a ‘polypill’ medication (comprising an angiotensin converting enzyme inhibitor, a beta-blocker, a calcium channel blocker, and a statin) in the prevention of cardiovascular disease (CVD). This intervention was selected as it has been flagged as an intervention of potentially significant benefit for the Indigenous population, and may assist in narrowing the health gap [26-28]. This intervention was evaluated as part of the ACE-Prevention project, however although the original intervention was specified for evaluation from mainstream GP settings, no specific data was available for its delivery via ACCHSs.

In this example, the two alternative target populations are the total general Australian population, and total Indigenous Australian population. In both cases, the intervention is applied to those aged 55 years and above, an age group considered to be at higher cardiovascular risk. For simplicity, differences according to remoteness have not been included. As this medication remains experimental, a hypothetical cost of AUD $500 per annum has been applied. This cost was the upper limit of a range considered reasonable by experts in the field for the cost of such a preparation [personal communication]. The upper limit was selected for this example as lower drug costs result in dominance of the intervention (health gain with cost-savings) when delivered from mainstream GP practices. Subsequently, when subject to economic evaluation the cost-effectiveness ratio is negative. The magnitude of negative cost-effectiveness ratios cannot be meaningfully compared across interventions, and thus is less informative as an example in this case. The intervention is evaluated as being delivered to the general Australian population via mainstream GP practices, and for the Indigenous population, has been evaluated as if delivered either via mainstream GP practices or ACCHSs (using the IHSD Template from Table 3).

The values of the different intervention parameters are shown in Table 4. The biological effectiveness of the intervention is assumed to be the same for both the general Australian and Indigenous populations (taken from the multiplicative effects of the component drugs from several studies [29-31]). It can be seen from Table 4 that intervention costs are the same if delivered via mainstream GP practices to both the general Australian and Indigenous populations, however there are differences in the utilization and treatment adherence rates, and cost-offsets between the two groups. If the intervention is delivered to the Indigenous population via ACCHSs, then the costs of the intervention are increased in line with the IHSD Template values while utilization and treatment adherence rates are also increased accordingly. An assumption has been made that 65% of eligible GPs participate in any particular intervention.

**Table 4:** Intervention parameters for the polypill intervention (Year 1 shown only)

**Intervention parameter**	**General population**	**Indigenous population**	
	**Mainstream GP services**	**Mainstream GP services**	**ACCHSs**
**Intervention effectiveness:**			
·RR Acute coronary syndrome	0.45	0.45	0.45
·RR stroke	0.31	0.31	0.31
**Intervention costs AUD$ (Yr 1):**			
·1 Long consultation	$58.55	$58.55	$155.78*
·2 Short consultations	$61.70	$61.70	$226.36*
·Medication	$500.00	$500.00	$500.00
·Pathology tests	$43.62	$43.62	$50.60* ($43.62 x 1.16)
**Health service utilization rate**	82%	49.2%* (82% x 60%)	60%* (82% x 73.2%)
**Treatment adherence rate**	60%	46.7%* (60% x 77.8%)	57.4%* (60% x 95.7%)
**Cost offsets AUD$ (Yr 1)** (savings per CVD case averted)	$11,078	$13,183* ($11,078 x 1.19)	$13,183* ($11,078 x 1.19)

* Indicates values for which the IHSD Template (from Table 3) has been applied (calculations in parentheses).

The values obtained in Table 4 were inputted into the relevant decision-analytic epidemiological models (modeling the prevention of CVD in either the general Australian or Indigenous populations) to perform the economic evaluations. The results of these evaluations are shown in Table 5.

**Table 5:** Cost-effectiveness of the polypill priced at $500 in preventing CVD

**Target population**	**Health service type**	**DALYs saved**	**Net costs (AUD$ millions)**	**Cost-effectiveness ratio (AUD$/DALY)**
Total Australian aged 55+	Mainstream GP practice	1000000	12000	11000
Total Indigenous aged 55+	Mainstream GP practice	550	7.2	13000
Total Indigenous aged 55+	ACCHS	830	17	21000

The results in this example reveal that the cost-effectiveness ratio is lowest for the intervention applied to the general Australian population via mainstream GP practices, and highest for the Indigenous population when delivered via ACCHSs. The smaller absolute number of DALYs saved and net costs for the Indigenous population are primarily due to the relatively smaller population size, but also reflect lower utilization and adherence rates compared to the general Australian population. Improved access for the Indigenous population results in a greater number of DALYs saved when the intervention is delivered via ACCHSs compared to mainstream GP practices, however the higher costs of running these services is reflected in greater net costs. The significance of these results is considered in the following discussion.

## Discussion

The Indigenous Health Service Delivery Template described in this paper provides a mechanism by which intervention data obtained from mainstream sources can be adapted to allow its economic evaluation as if delivered from an Indigenous setting, when Indigenous specific evidence is deficient. Thereby, results are made more relevant to the Indigenous context.

The results reveal that the cost of a health practitioner encounter (or consultation) at an ACCHS is more expensive than a consultation at a mainstream GP practice, due to the additional components identified as part of providing the ACCHS model of comprehensive primary health care (Table 3). This contributes to the greater net costs of interventions; in the case of the polypill example, AUD $17 million when delivered via ACCHSs compared to $7.2 million from mainstream GP practices (Table 5), an increase of 58%. Health service provision from ACCHSs is also more expensive in remote regions compared to non-remote areas due to the additional services involved. Cost-offsets are higher for the Indigenous population irrespective of what type of health service is accessed due to the greater costs associated with treating more complex disease in this population [32].

One outcome of these differing models of health care is that health service access can be improved for Indigenous Australians. This is illustrated by higher Indigenous rates of utilization of health services and adherence to treatments delivered from ACCHSs compared to mainstream GP practices. Therefore the health benefits of interventions are also greater, with the polypill example revealing 50% more net benefit when the intervention is delivered from ACCHSs compared to mainstream GP practices (830 compared to 550 DALYs saved from Table 5). Such improvements are important in addressing health inequities and attempts to narrow the Indigenous health gap.

The overall impact of these differences in costs and effects is that despite their greater benefits, cost-effectiveness ratios are higher if interventions are delivered to the Indigenous population via ACCHSs compared to mainstream GP practices (in the case of the polypill example, $21,000 per DALY prevented compared to $13,000 per DALY prevented respectively). This is because the additional costs involved in the delivery of interventions from ACCHSs are proportionally greater than the additional benefits. When analysis is restricted to interventions delivered from mainstream GP practices, it is more cost-effective (lower cost-effectiveness ratio) to deliver the intervention to the general Australian population rather than the Indigenous population (for the polypill, $11,000 compared to $13,000 DALY/saved). This can be attributed to differences in adherence rates between the two populations. Similar overall results were obtained in the economic evaluation of several other case study interventions performed as part of the ACE-Prevention project [33]. The need to interpret these results carefully and with reference to context is discussed in the following section.

### Implications of results

From the development of the IHSD Template, it can be seen that delivery of interventions via ACCHSs provides greater health benefits for the Indigenous population compared to delivery via mainstream GP practices. However, these benefits require additional resources and therefore come at greater cost. Use of the template in an example economic evaluation has revealed a higher cost-effectiveness ratio when an intervention is delivered to the Indigenous population from ACCHSs compared to delivery from mainstream GP practices. Moreover, results indicate it is more cost-effective to apply interventions to the general Australian rather than the Indigenous population from mainstream GP settings. However, these results require careful analysis.

It is clear that simplistic interpretation of cost-effectiveness ratios in this case would direct resources away from ACCHSs and the Indigenous population in general, and is therefore unlikely to be acceptable on social justice grounds. In light of the present Indigenous health disadvantage and the stated Australian government policy goal of bridging the health gap, the economic question instead becomes whether the additional spend on Indigenous health represents a worthwhile use of resources. In other words, is the additional health benefit achieved with the provision of interventions via ACCHSs worth the additional costs? The answer not only relates to efficiency, but in the case of disadvantaged groups, concerns for equity and social justice are often paramount. These considerations should be borne in mind when assessing cost-effectiveness ratios, for example, via the application of equity weights. It is not the intention to examine these issues within this discussion, however the topic has been addressed by the authors in a separate paper [34]. It is important that these results contribute to decision-making, but are not considered in isolation from the broader policy factors.

### Advantages of the IHSD Template

One of the main advantages of the IHSD Template is that it provides a simple and convenient mechanism by which the economic evaluation of interventions can be made more relevant to the Indigenous population when Indigenous specific evidence is not available. Therefore economic evaluation results are made more applicable to Indigenous health, and can provide valuable evidence to assist resource allocation decision-making within this context. In addition, the IHSD Template can assist in quantifying and valuing the amount of resources required to deliver interventions equitably to the Indigenous population using best practice mechanisms, and thereby contribute to determining funding allocations.

### Limitations of the IHSD Template

Measurement and valuation of IHSD Template components has required extrapolation from data sources which may not precisely represent those components, and due to resource constraints, interviews performed provided informative rather than comprehensive data. Therefore, this research should be seen as a scoping exercise in the development of a prototype IHSD Template and is not prescriptive. These results nevertheless may serve as a guide to health service requirements, and can indicate areas where data gaps exist and further research is required.

It is acknowledged that the method of comparing the additional components provided in the delivery of health interventions via ACCHSs with delivery via mainstream GP services could be considered simplistic and to not adequately capture the complexity and interconnectedness of an Indigenous health service [19]. However, in light of the overall lack of such evidence, the IHSD Template could be deemed an appropriate starting point that provides improved estimates over results based purely on mainstream evidence alone.

Other potential limitations of the technique include that the application of additional costs associated with ACCHSs delivery to a ‘patient encounter’ means only interventions which include health practitioner consultations can be evaluated using the IHSD Template. In addition, the IHSD Template is a broad generalization of what are inherently context specific health services, although some differentiation has been made for services provided in remote and non-remote areas. The specificity of the template needs to be balanced against the amount of data required in its construction and its ease of use in practice. Further qualitative judgements are required as part of the decision-making process to take these factors into account.

### Implications of the IHSD Template for policy and practice

When using economic evaluation results to inform resource allocation decision-making, it is important that analyses are relevant to the target populations to whom the interventions are applied. This research has illustrated that there can be large differences in how a particular intervention is delivered between different health service delivery models, and this impacts on both the costs of the intervention and its effectiveness. As a consequence, the economic evaluation results for a single intervention can differ according to the target population and health service context in which it is delivered, and it is not appropriate to assume that cost-effectiveness ratios are the same for all population sub-groups.

The lack of intervention cost and effectiveness data for minority disadvantaged groups, such as Australia’s Indigenous population, on which to base economic evaluations is inevitable in light of limited research capacity and evaluation budgets. Yet economic evidence remains an important tool in prioritizing the need for interventions aimed at improving health inequities. The IHSD Template proposed in this paper enables evidence to be made more relevant to the Indigenous population without the need to specifically trial every intervention from an Indigenous health service context. The method is also generalisable to other population sub-groups who experience health disadvantage, and for whom an equitable model of health service delivery can be identified.

On a broader scale, the results of this analysis illustrate the potential benefits of targeted models of health service delivery over mainstream health services in addressing health inequities. The same interventions can produce more health gain for Australia’s Indigenous population if delivered from ACCHSs compared to mainstream GP services, and thus provide a valuable mechanism in themselves by which the Indigenous health gap could be addressed, irrespective of the types of interventions implemented. However, these advantages come at greater cost and additional resources are required.

These results have implications for how health services are optimally funded. In Australia, ACCHSs tend to be funded from multiple sources via project specific grants [14]. However, a more appropriate alternative would be holistic funding of these health services in recognition of their inherent benefits as a health care delivery vehicle (over and above the benefit of any interventions delivered from them). Via the identification, measurement and valuation of the additional components provided as part of ACCHS care, the IHSD Template could indicate the amount of resources required to provide health services equitably, and further research in this direction is warranted. In this way, interventions could subsequently be selected by health services which are culturally sensitive and in line with their budgets and locally determined health needs.

## Conclusion

This paper has described the development and application of the Indigenous Health Service Delivery (IHSD) Template; a tool for use in the economic evaluation of primary health care interventions targeting the Australian Indigenous population when context specific data is deficient. The template identifies, measures and values differences in the delivery of health interventions between ACCHSs and mainstream GP practices. This then allows intervention cost and effectiveness data from mainstream to be adapted to allow its economic evaluation is *as if* delivered from an Indigenous setting. The method is simple in its application, and reveals that more resources are required in the provision of interventions from ACCHSs. This is associated with greater health benefits due to improved service access, and these changes impact on the calculated cost-effectiveness ratios that ensue.

The approach highlights the importance of access to context specific health economics data when assessing interventions for disadvantaged groups. Failure to do so may result in resource allocation decisions based on evidence which is not representative, and this could perpetuate or even exacerbate health inequities. Although the example of Australia’s Indigenous population has been used, the technique could be generalized to other minority groups who experience health disadvantage and for whom an equitable model of health service delivery is available. This is particularly important if addressing health inequities is a stated policy goal.

## Abbreviations

ACCHS: Aboriginal community controlled health service; ACE: Assessing cost effectiveness; CVD: Cardiovascular disease; DALY: Disability adjusted life year; GP: General practitioner; IHSD: Indigenous health service delivery.

## Competing interests

IA, although having no financial interest, has previously serviced on the board of an Aboriginal Health Service. The other authors declare that they have no competing interests.

## Authors’ contributions

KO, IA and RC conceptualized the study. KO designed the study with input and coordination from IA, RC and MK. KO and IA developed the data collection methods. KO piloted the data collection methods and collected the data. KO drafted the manuscript. All authors read and approved the final manuscript.

## Authors’ information

KO has completed a PhD at the School of Population Health, The University of Melbourne. RC has a continuing appointment as Professor of Health Economics at Deakin University, and was a Chief Investigator on the ACE-Prevention project. Associate Professor MK holds and ARC Future Fellowship at the School of Population Health, The University of Melbourne. IA is Professor and Director, *Murrup Barak* – Melbourne Institute for Indigenous Development, The University of Melbourne.

## Pre-publication history

The pre-publication history for this paper can be accessed here:

http://www.biomedcentral.com/1472-6963/12/307/prepub

## Supplementary Material

Additional file 1 **Measurement and valuation of Indigenous Health Service Delivery Template components.** A table providing more detailed information on the calculation of the IHSD Template values and the sources used. Click here for file
