# Supplementary material for: Differences in primary health care delivery to Australia’s Indigenous population: a template for use in economic evaluations
Source: BMC Health Serv Res. 2012 Sep 7;12:307. doi: 10.1186/1472-6963-12-307 (PMC3468365; doi:10.1186/1472-6963-12-307)
Supplement: Additional file 1 — Measurement and valuation of Indigenous Health Service Delivery Template components. A table providing more detailed information on the calculation of the IHSD Template values and the sources used. [file 1472-6963-12-307-S1.pdf]

Additional file 1

Measurement and valuation of Indigenous Health Service Delivery Template components

Intervention delivery via Aboriginal Community Controlled Health Services compared to mainstream general practitioner services, combined across all remoteness locations

Component	Quantity (measurement)	Cost (value)	Additional cost per patient encounter	Sources
Cost of consultation in mainstream GP practices	-	$30.85 short consult, $58.55 long consult	-	Medicare Benefits Schedule 2003 [1]
**Basic health intervention delivery characteristics**				
Role substitution	Proportional substitution of general practitioner with Aboriginal health worker or nurse<br>Longer average consultation lengths associated with different practitioners	Composite wage rate	$11.75 per short consultation (including on-costs)<br>$26.65 per long consultation	Service Activity Reports (SARs) data 2003-04 [2]<br>Econtech Pty Ltd, 2004 [3]<br>Interviews
Compliance management	1.5 hrs/wk per full time staff member	Nurse wage rate	$2.13	SARs data 03-04 [2]<br>Econtech Pty Ltd, 2004 [3]<br>Interviews
Home visits	10% of consults cf. 1.8% in mainstream	$21.10 per home visit	$2.79	Larkins, 2006 [4]<br>Thomas, 1998 [5]<br>MBS 2003 [1]<br>Interviews
Paperwork/Case conferencing	Equivalent to mainstream	-	-	Interviews
Training activities	Equivalent to mainstream	-	-	Interviews
Management of complex medical conditions	Included in longer consultation lengths	-	-	Interviews
Opportunistic screening of family members	Included in total number of patient consultations in health service	-	-	Interviews
**Population health, social and community activities**				
Other consultative services	36%of all urban consults, 23% rural, 15% remote	Composite allied health wage rate	$9.28 (including on-costs)	SARs data 03-04 [2]<br>Econtech Pty Ltd, 2004 [3]<br>Interviews
Service substitution	Not costed in study	-	-	No adequate source found

Health promotion/health education	Included in longer consultation lengths	-	-	Sibbald, 2006 [6]
Community development	Included under 'community management boards' component	-	-	Interviews
Community space	-	Not costed as does not incur significant health service cost	-	Interviews
**Administration, management and governance structures**				
Overheads	30% loading for existing staff<br>60% loading for new and remote staff	Composite wage rate	Included in 'role substitution' component	ACE-Prevention economic protocol [7]
Community management boards	Ne board per service	$4000 per annum administrative expenses	$0.29	Victorian Department of Human Services [8]
Management, Economic, Social, Human (MESH) infrastructure<br>(Management capacity building)	Currently excluded from analysis as considered a start-up cost and not in line with 'steady state' assumption of analysis	-	-	Henry, 2003 [9]<br>Houston, 2003 [10]<br>ACE-Prevention economic protocol [7]
Additional management activities	80% full time equivalent management position	Management wage rate	$3.58	Interviews<br>Australian Industrial Relations Commission, 2005 [11]
**Patient transport services**				
Patient transport	50% patients cf. 7% in mainstream<br>Distance: 20km urban areas cf. 32km in mainstream	$2.12 per km cf. $0.75 in mainstream	$47.01	NSW Transport Planning and Management and Applied Economics, 2001 [12]<br>SARs data 03-04 [2]<br>Interviews
**Provision of services to a large remote population**				
Out of hours emergency care	52% remote ACCHSs<br>Emergency equipment<br>20 hours overtime per service per week<br>Annual staff training	$3900 cardiac defibrillator<br>Composite wage rate<br>$2000 training fee	$1.31	Rural Ambulance Victoria, 2007 [13]<br>Australian Industrial Relations Commission [14-16]<br>Mole, 2006 [17]<br>O'Meara, 2002 [18]
Outreach services	4 weekly visits of 9 hours duration	Composite wage rate	$2.30	Battye, 2003 [19]<br>SARs data 03-04

	2.5 staff<br>60% on-costs<br>Average 2.9 sites 170 km from main service	$2.15 per km travelled		[2]<br>Interviews
Staff housing and relocation	Housing for 4 staff per service<br>1 relocation grant 2 yearly	Housing $219 per week<br>Relocation grant $20,000	$1.89	Australian Rural and Remote Workforce Agencies Group, 2007 [20]<br>Realestate.com.au [21]
Pharmaceuticals and pathology	n/a	60% pathology cost loading in remote areas	n/a	Interviews
**Differences in Indigenous utilisation and adherence**				
Indigenous rates of utilisation of health services	Indigenous utilisation of mainstream services 60% that of non-Indigenous based on BEACH (Bettering the Evaluation and Care of Health) data<br>22% improvement in Indigenous utilisation with culturally appropriate services based on local evaluation, therefore 73% of non-Indigenous utilisation rate in ACCHSs	n/a	n/a	Pink, 2008 [22]<br>Katherine West CCT evaluation [23]
Indigenous rates of adherence to treatment regimens	Indigenous adherence in mainstream 78% that of non-Indigenous based on differing rates of vaccination coverage<br>23% improvement in vaccination rates in very remote areas compared to urban areas possibly due to presence of targeted services. Equates to Indigenous adherence in ACCHSs 96% that of non-Indigenous population	n/a	n/a	Hull, 2004 [24]<br>Haynes, 2004 [25]
**Cost offsets**				
Cost offsets	n/a	Cost-offsets based on average case-adjusted inpatient treatment costs	1.19 ratio between Indigenous and non-Indigenous populations	Fisher, 1998 [26]

References

1. Department of Health and Ageing, Australian Government: **Medicare Benefits Schedule Book.** Commonwealth of Australia, Canberra; 2003.

2. Office for Aboriginal and Torres Strait Islander Health, National Aboriginal Community Controlled Health Organisation: **Service Activity Reporting: 2003-04 Key Results.** Canberra; 2006.

3. Econtech Pty Ltd: **Costing models for Aboriginal and Torres Strait Islander health services.** Commonwealth of Australia,, Canberra; 2004.

4. Larkins SL, Geia LK, Panaretto KS: **Consultations in general practice and at an Aboriginal community controlled health service: do they differ?** *Rural Remote Health* 2006, **6:**560.

5. Thomas DP, Heller RF, Hunt JM: **Clinical consultations in an aboriginal community-controlled health service: a comparison with general practice.** *Aust N Z J Public Health* 1998, **22:**86-91.

6. Sibbald B, Laurant MG, Reeves D: **Advanced nurse roles in UK primary care.** *Med J Aust* 2006, **185:**10-12.

7. Carter R, Vos T, Barendregt J: **ACE-Prevention Economic Evaluation Protocol.** Deakin University, Melbourne and the University of Queensland, Brisbane; 2005.

8. Department of Human Services: **Appointments to Community Health Centre Boards of Management.** State Government of Victoria, Melbourne; 2007.

9. Henry BR, Mooney GH: **Improving Aboriginal health: more than just chucking more money at it. SPHERe report in primary health care funding of Aboriginal health.** Social and Public Health Economics Research Group, Curtin University of Technology; 2003.

10. Houston S: **The past, the present, the future of Aboriginal health policy.** *PhD Thesis.* Curtin University of Technology, Division of Health Sciences; 2003.

11. Australian Industrial Relations Commission: **AP843641 - Health, Community Services and Ambulance - Management and Administrative Staff (Public Sector - Victoria) Award 2005.** 2005.

12. Transport Planning and Management and Applied Economics: **Non-emergency health-related transport - Facilitating access to health services in NSW.** Sydney; 2001.

13. Rural Ambulance Victoria: **Remote Area Nurse Training.** 2007.

14. Australian Industrial Relations Commission: **Aboriginal and Community Controlled Health Services (Community Health Nursing Staff) Award 2002.** 2007.

15. Australian industrial Relations Commission: **Aboriginal Medical Service Employees' Award.** 2005.

16. Australian Industrial Relations Commission: **Medical Officers (Aboriginal Medical Services) Interim Award 2001.** 2006.

17. Mole J, McGuire D, Riley R, Trewhella N: **Are Perth metropolitan GPs prepared for common medical emergencies?** *Aust Fam Physician* 2006, **35:**553-554.

18. O'Meara P, Burley M, Kelly H: **Rural urgent care models: what are they made of?** *Aust J Rural Health* 2002, **10:**45-50.

19. Battye KM, McTaggart K: **Development of a model for sustainable delivery of outreach allied health services to remote north-west Queensland, Australia.** *Rural Remote Health* 2003, **3:**194.

20. Australian Rural and Remote Workforce Agencies Group: **Supports available to current and future GPs in rural and remote Australia.** [Online] Available at: http://www.arrwag.com.au/client_images/234218.doc [Accessed 4th July 2007].

21. **realestate.com.au** [http://www.realestate.com.au/]

22. Pink B, Allbon P: **The health and welfare of Australia's Aboriginal and Torres Strait Islander Peoples.** Australian Bureau of Statistics, Australian Institute of Health and Welfare; 2008.

23. Miyrta J: **Katherine West Coordinated Care Trial: Local Evaluation Final Report.** Menzies School of Health Research; 2000.

24. Hull BP, McIntyre PB, Couzos S: **Evaluation of immunisation coverage for aboriginal and Torres Strait Islander children using the Australian Childhood Immunisation Register.** *Aust N Z J Public Health* 2004, **28:**47-52.

25. Haynes K, Stone C: **Predictors of incomplete immunisation in Victorian children.** *Aust N Z J Public Health* 2004, **28:**72-79.

26. Fisher DA, Murray JM, Cleary MI, Brewerton RE: **The Aboriginal and Torres Strait Islander casemix study.** *Med J Aust* 1998, **169 Suppl:**S11-16.
